# Supplementary material for: The microbiome of cryospheric ecosystems
Source: Nat Commun. 2022 Jun 2;13:3087. doi: 10.1038/s41467-022-30816-4 (PMC9163120; doi:10.1038/s41467-022-30816-4)
Supplement: Supplementary file 1 — Supplementary information [file 41467_2022_30816_MOESM1_ESM.pdf]

Supplementary information:

The microbiome of cryospheric ecosystems

Massimo Bourquin[1,*, $], Susheel Bhanu Busi[2,*], Stilianos Fodelianakis[1], Hannes Peter[1], Alex Washburne[3], Tyler J. Kohler[1], Leïla Ezzat[1], Grégoire Michoud[1], Paul Wilmes[2], Tom J. Battin[1,$]

[1] River Ecosystems Laboratory, Centre for Alpine and Polar Environmental Research (ALPOLE), École Polytechnique Fédérale de Lausanne, EPFL, Switzerland

[2] Luxembourg Centre for Systems Biomedicine, University of Luxembourg, University of Luxembourg, Campus Belval, 7, avenue des Hauts-Fourneaux, L-4362 Esch-sur-Alzette, Luxembourg

[3] Selva Analytics LLC, Bozeman, MT, 59718, USA

[4] Department of Life Sciences and Medicine, Faculty of Science, Technology and Medicine, University of Luxembourg, 7, avenue des Hauts-Fourneaux, L-4362 Esch-sur-Alzette, Luxembourg

*Equal contributions

[$]Correspondence to: MB (massimo.bourquin@epfl.ch) and TJB (tom.battin@epfl.ch)

Content:

- Page 2-5: Supplementary Figures 1-4

- Page 6-14: Supplementary Tables 1-8

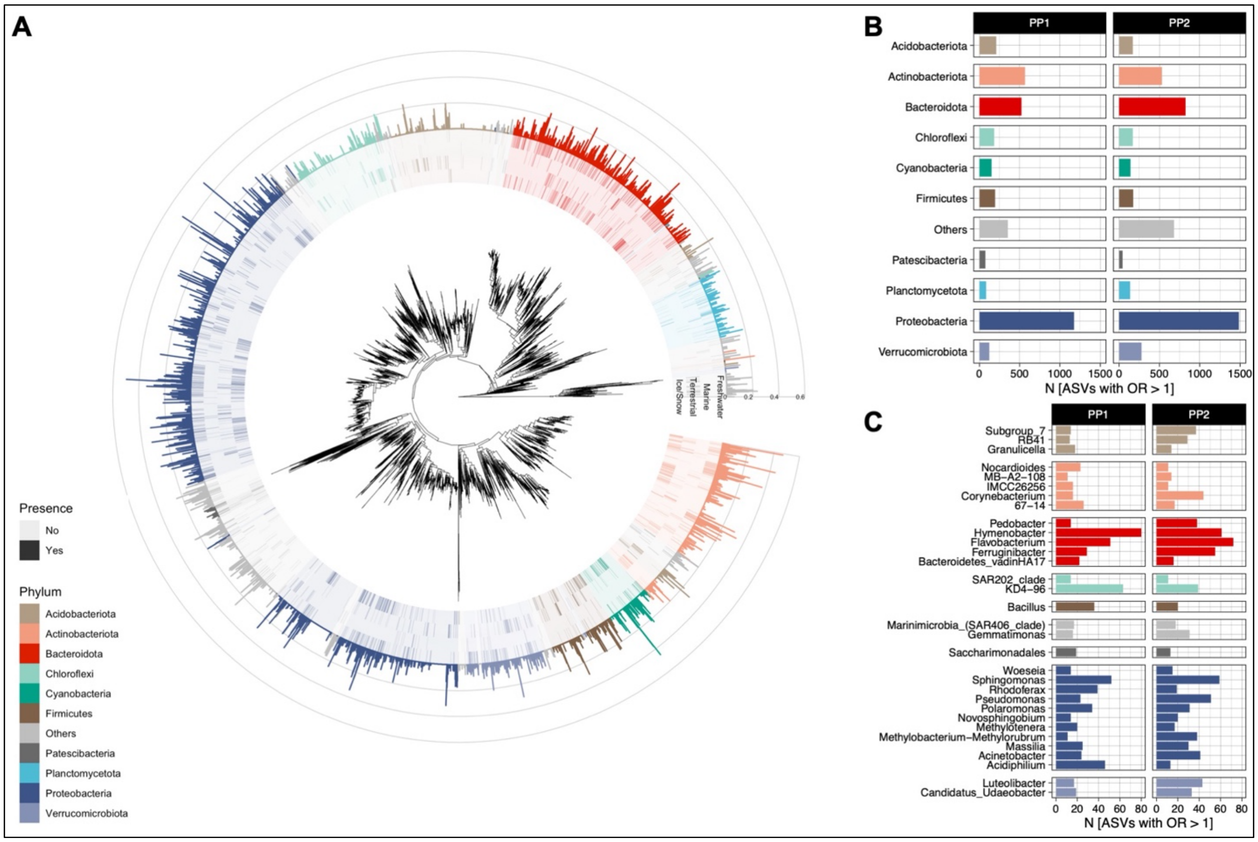

Supplementary Figure 1. A) Phylogenetic tree based on the abundant ASVs (relative abundance > 0.5%) found in PP2, demonstrating the cryosphere microbiome diversity across the bacterial tree of life. The highlighted colors represent the ASVs detected in cryospheric ecosystems, and the barplot represents the coefficient for the logistic classification analysis. The number of ASVs at **B)** the phylum-level, and **C)** genus-level taxonomy with an odds-ratio greater than 1 in the logistic classification is shown for PP1 and PP2. Only taxa with the highest numbers are shown.

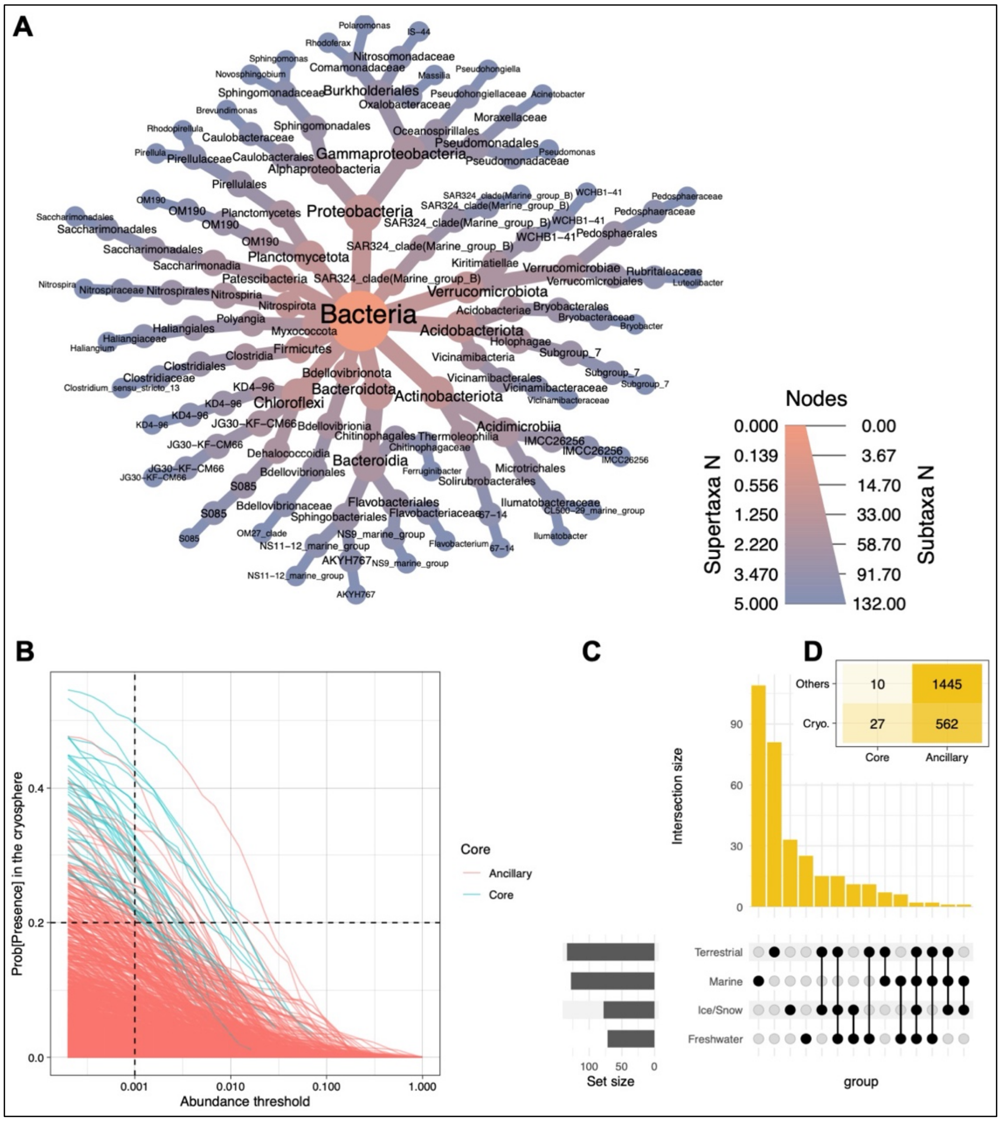

Supplementary Figure 2. A) Heat-tree showing the taxonomic classification of the 37 bacterial genera representing the core microbiome of the cryosphere (out of the 2044 total bacterial genera). They represent all bacterial genera with a probability of presence of 20% (calculated in the binomial model analysis, abundance threshold of 0.1%) in the cryosphere, and present in all four ecosystem types. **B)** Line graphs depicting the probability of presence of a given bacterial genus in the cryosphere and their respective abundance, to identify the 'core' (blue) and 'ancillary' (red) genera. The dashed lines represent the chosen thresholds for the core microbiome definition. **C)** Upset plot showing the overlap across the ecosystem types core microbiome, defined at the genus-level, with a prevalence of 20% at an abundance threshold of 0.1% relative abundance. **(D)** Heat plot showing the number of genera per group, highlighting the large overlap between the core microbiome and the cryospheric genera.

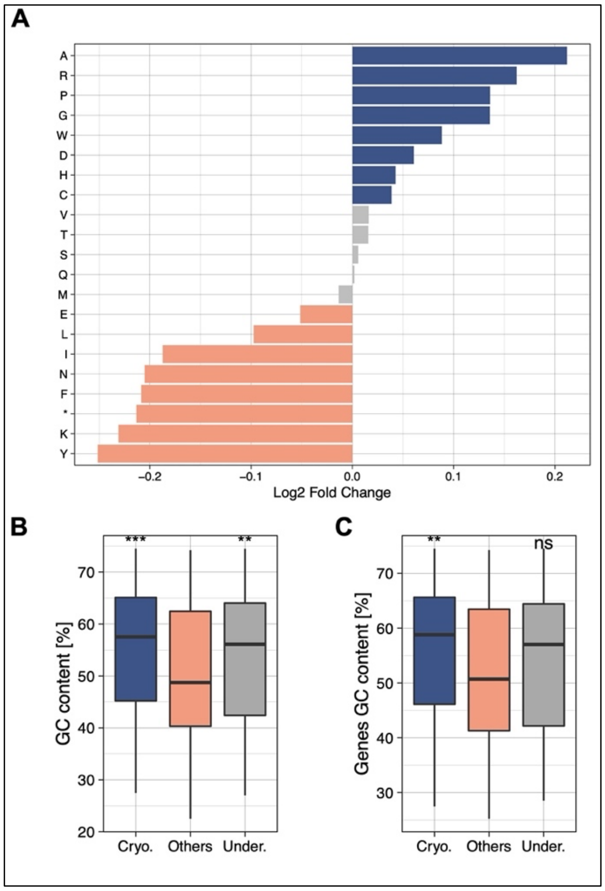

Supplementary Figure 3. A) The enriched abundance of aminoacids in the cryosphere (blue; positive fold change) compared to those found in the non-cryospheric ecosystems are depicted. The "*" represents stop codons. **B)** The overall GC% of the genomes belonging to the cryospheric, others and underrepresented genera are depicted (n = 660 total bacterial genera, 197 of which are cryospheric genera, 198 are underrepresented in the cryosphere). **C)** The GC% of the genes predicted in the genomes belonging to the cryospheric, others and underrepresented genera are depicted. The median, 25% and 75% quartiles are represented in the boxplots. Two-sided Wilcoxon tests were performed to assess significance in panels B and C; the Holm method was used to correct for multiple testing (***: 0-0.001, **: 0.001-0.01). Boxplots depict the median and the 25th and 75th quartiles, whiskers extend to values within 1.5 times the interquartile range, and the remaining points are outliers. Exact p-values and medians are listed in Supplementary Table 6.

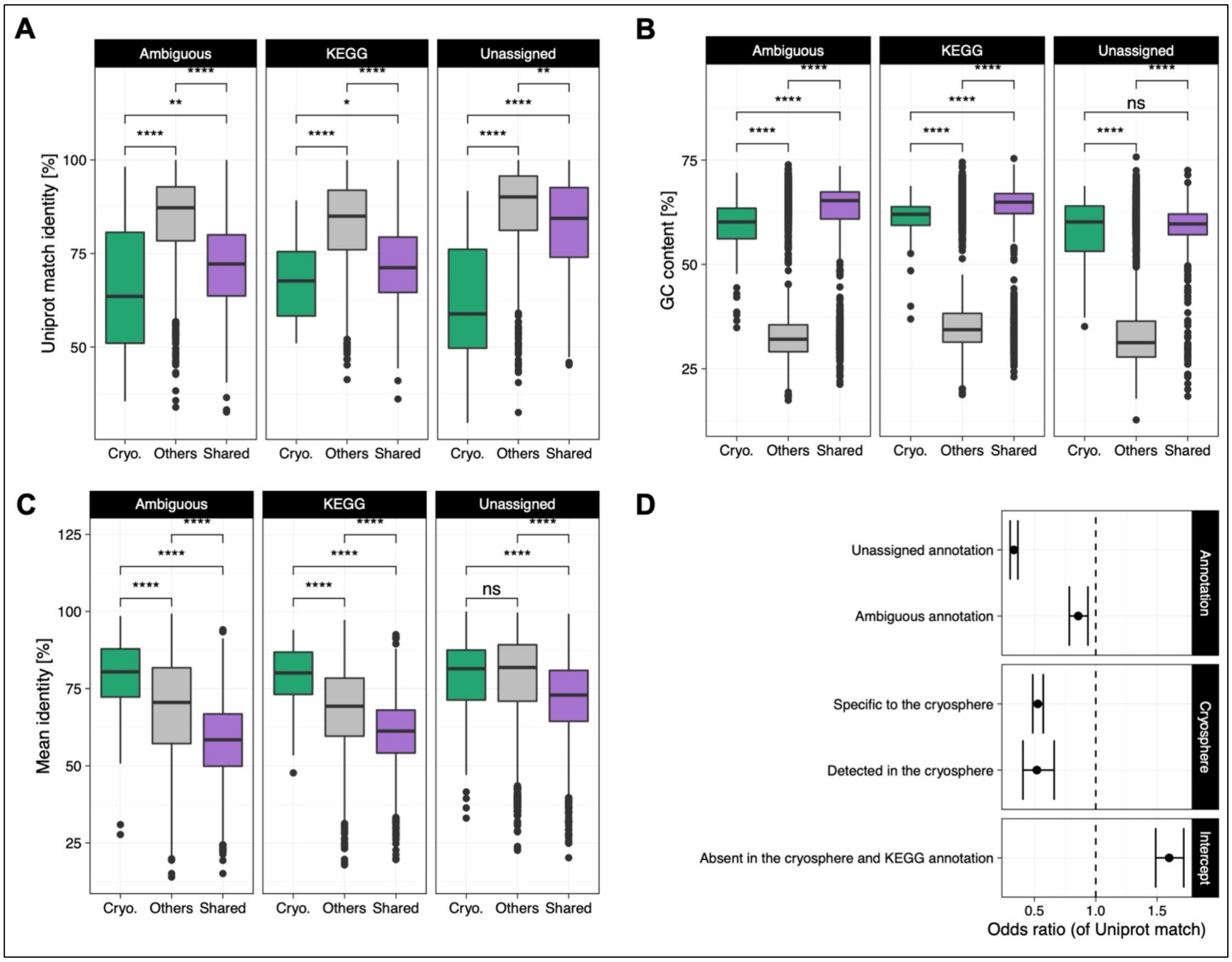

Supplementary Figure 4. A) Boxplots indicate the overall identity percentage for representative sequences for each of the gene clusters that matched with UniProt sequences. The figures demonstrate these values for the Cryosphere-only genes, non-cryosphere (Othersx) and those 'shared' between the two habitats. **B)** The GC content % of all the genes within each cluster is shown. **C)** The pairwise identity of all sequences within each cluster is represented on the boxplots. **D)** Odds-ratio estimations of the UniProt matches with respect to the annotation level, and the presence/or not in the cryospheric metagenomes. Two-sided Wilcoxon tests were performed to assess significance in panels A, B and C; the Holm method was used to correct for multiple testing (****: 0-0.0001, ***: 0.0001-0.001, **: 0.001-0.01, *: 0.01-0.05). Boxplots depict the median and the 25th and 75th quartiles, whiskers extend to values within 1.5 times the interquartile range, and the remaining points are outliers. Sample sizes are listed in Table 1. The exact p-values and test statistics are available in the Supplementary Table 8.

Dataset	Accuracy [%]	Precision [%]	Recall [%]	AUC [%]
PP1	96.04±1.93	99.99±0.05	92.08±3.86	99.93±0.02
PP2	97.95±1.35	99.93±0.14	95.96±2.71	99.93±0.02

Supplementary Table 1. Cryospheric bacterial communities' logistic classification models performance summary for each primer pair dataset.

Dataset	Group 1	Group 2	Sorensen's index			ß-MNTD		
			Corrected p-value	r	Median difference	Corrected p-value	r	Median difference
**PP1**	Cryo-Cryo	Cryo-Others	< 2e-16	0.185	0.038	< 2e-16	0.0821	0.012
	Cryo-Cryo	Other-Others	< 2e-16	0.140	0.042	< 2e-16	0.0639	0.01
	Cryo-Others	Other-Others	7.6e-13	0.0174	0.004	< 2e-16	0.144	0.022
**PP2**	Cryo-Cryo	Cryo-Others	< 2e-16	0.238	0.046	<2e-16	0.183	0.032
	Cryo-Cryo	Other-Others	< 2e-16	0.263	0.057	<2e-16	0.125	0.028
	Cryo-Others	Other-Others	< 2e-16	0.0501	0.011	<2e-16	0.0503	0.004

Supplementary Table 2. ß-diversity phylogenetics (Sorensen's Index and ß-MNTD) computed for 50 iterations randomly drawing 50 cryospheric and 50 non-cryospheric samples (sample sizes for each group: $N_{PP1\text{-}Sor}$ = 83583, $N_{PP2\text{-}Sor}$ = 98142, $N_{PP1\text{-}MNTD}$ = 77893, $N_{PP2\text{-}MNTD}$=91398). For both datasets and primer pairs, the Kruskal-Wallis tests were highly significant (p-value < 2.2e-16), post-hoc two-sided Wilcoxon tests results are reported in the table, the p-value was corrected using the Holm method. The effect size was computed as r with the *statix* R package.

		α-MPD (model p-value: < 2.2e-16)		
		**Estimate**	**p-value**	**t-value**
**Coefficients**	**Intercept**	0.389±0.014	28.667	<2e-16
	**Cryosphere**	0.077±0.005	13.933	<2e-16
	**log(SR)**	0.062±0.004	15.973	<2e-16
	**DatasetPP2**	-0.038±0.004	-9.129	<2e-16
**Model**	**Adj. R^2**	0.108		
	**df**	4240		
		**α-MNTD (model p-value: < 2.2e-16)**		
		**Estimate**	**t-value**	**p-value**
**Coefficients**	**Intercept**	0.307±0.006	48.933	<2e-16
	**Cryosphere**	0.015±0.003	5.734	1.05e-08
	**log(SR)**	-0.053±0.002	-29.399	<2e-16
	**DatasetPP2**	0.020±0.002	10.302	<2e-16
**Model**	**Adj. R^2**	0.191		
	**df**	4240		
		**α-PD (model p-value: < 2.2e-16)**		
		**Estimate**	**t-value**	**p-value**
**Coefficients**	**Intercept**	0.450±0.047	9.483	<2e-16
	**Cryosphere**	0.532±0.049	10.955	<2e-16
	**SR**	0.110±0.001	89.696	<2e-16
	**DatasetPP2**	0.398±0.037	10.887	<2e-16
**Model**	**Adj. R^2**	0.664		
	**df**	4240		

Supplementary Table 3. α-diversity phylogenetics (MPD: mean phylogenetic distance, MNTD: mean nearest taxon distance; PD: phylogenetic diversity) linear models testing the influence of the cryosphere on the different metrics, with the species richness (SR, log-transformed) and the dataset as fixed effects (Intercept = non-cryospheric, and PP1).

**PERMANOVA**	**DF**	**Sum of squares**	**r^2**	**f-value**	**p-value**
Ecosystem	3	55.774	0.18319	52.702	< 0.001
Dataset	1	5.633	0.01850	15.969	< 0.001
Residual	689	243.055	0.79831		
Total	693	304.462	1.00000		

**Snow/Ice – Terr.**	**DF**	**Sum of squares**	**r^2**	**f-value**	**p-value**
Ecosystem	1	18.859	0.11779	54.36	< 0.001
Dataset	1	4.906	0.03064	14.14	< 0.001
Residual	393	136.340	0.85157		
Total	395	160.105	1.00000		

**Snow/Ice – Marine**	**DF**	**Sum of squares**	**r^2**	**f-value**	**p-value**
Ecosystem	1	23.280	0.15758	66.918	< 0.001
Dataset	1	4.087	0.02767	11.749	< 0.001
Residual	346	120.370	0.81476		
Total	348	147.737	1.00000		

**Snow/Ice – Fresh.**	**DF**	**Sum of squares**	**r^2**	**f-value**	**p-value**
Ecosystem	1	10.991	0.06768	28.6254	< 0.001
Dataset	1	3.195	0.01967	8.3202	< 0.001
Residual	386	148.211	0.91265		
Total	388	162.397	1.00000		

**Marine – Terr.**	**DF**	**Sum of squares**	**r^2**	**f-value**	**p-value**
Ecosystem	1	25.402	0.20705	85.019	< 0.001
Dataset	1	7.052	0.05748	23.604	< 0.001
Residual	302	90.230	0.73547		
Total	304	122.684	1.00000		

**Marine – Fresh.**	**DF**	**Sum of squares**	**r^2**	**f-value**	**p-value**
Ecosystem	1	18.464	0.14665	53.295	< 0.001
Dataset	1	5.240	0.04162	15.125	< 0.001
Residual	295	102.202	0.81173		
Total	297	125.906	1.00000		

**Terrestrial - Fresh.**	**DF**	**Sum of squares**	**r^2**	**f-value**	**p-value**
Ecosystem	1	16.200	0.11536	47.332	< 0.001
Dataset	1	7.175	0.05109	20.963	< 0.001
Residual	342	117.056	0.83355		
Total	344	140.431	1.00000		

Supplementary Table 4. Model summaries for the PERMANOVA and all pairwise.adonis comparisons of cryospheric ecosystem types.

**WTS model**	**Test statistic**	**df**	**p-value WTPS**	
Ecosystem	112.0236	3	0	
Interaction	103.1681	4	0	
**Ecosystem**	**Datastet**	**N**	**Mean**	**Variance**
Freshwater	PP1	29	3.72	0.26
Freshwater	PP2	140	2.84	0.48
Ice/Snow	PP1	92	2.76	0.69
Ice/Snow	PP2	128	2.93	0.68
Marine	PP1	88	3.03	0.18
Marine	PP2	41	3.71	0.41
Terrestrial	PP1	92	3.69	0.80
Terrestrial	PP2	84	3.64	0.28

Supplementary Table 5. Shannon's index H α-diversity (calculated at the genus taxonomic level) Wald-Type Statistic (WTS) summary. This test was chosen as a non-parametric alternative to ANOVA for non-normally distributed data. "Interaction" represents the interaction between the Ecosystem and Dataset parameters, "Ecosystem" the fixed effect of the ecosystem type. The computed means weighted by sample sizes are 2.987, 2.856, 3.245, and 3.669 for freshwater, snow/ice, marine and terrestrial, respectively.

Metric	$\textbf{Median}_{\textbf{Others (n=265)}}$	$\textbf{Median}_{\textbf{Cryo (n=197)}}$	$\textbf{Median}_{\textbf{Under.(n=198)}}$
GC content [%]	48.7	57.5 (corr. p = 0.0011)	56.1 (corr. p = 0.0086)
Genome size [mbp]	3.97	4.19 (corr. p = 0.17)	4.06 (corr. p = 0.28)
Growth doubling time [d]	4.48	4.12 (corr. p = 0.87)	4.55 (corr. p = 0.51)
Codon usage bias [CUBHE]	0.627	0.627 (corr. p = 1)	0.623 (corr. p = 1)
Consistency [HE]	0.527	0.519 (corr. p = 0.84)	0.529 (corr. p = 0.25)
Codon pair bias [CPB]	-0.375	-0.370 (corr. p = 0.92)	-0.380 (corr. p = 0.74)

Supplementary Table 6. RefSeq genomic properties summary. Corrected p-values were computed using two-sided Wilcoxon tests implemented in the *compare_means* function of the *ggpubr* R package, comparing the cryospheric and underrepresented genera against the others.

**Dataset**	**Study ID**	**Sample n.**	**Ecosystem type**	**Description**
PP1	Bergk2019	12	Ice/Snow	Snow samples, Svalbard
PP1	NOMIS	10	Freshwater	Glacier-fed stream sediment samples, Russia and New Zealand
PP1	PRJDB9246	11	Freshwater	Microbial mat and water, Antarctica
PP1	PRJEB12640	41	Terrestrial	Soil chronosequence samples, Svalbard
PP1	PRJEB26163	62	Marine	Marine water, Arctic Ocean
PP1	PRJEB29215	32	Ice/Snow	Snow samples, Antarctica
PP1	PRJEB31938	26	Marine	Sea ice, snow, water and sediment, Greenland and the Arctic Ocean
PP1	PRJEB40467	18	Terrestrial	Alpine permafrost, Italy
PP1	PRJNA296475	3	Ice/Snow	Cryoconite hole, Svalbard
PP1	PRJNA320505	1	Ice/Snow	Cryoconite hole, Antarctica
PP1	PRJNA380676	1	Terrestrial	Arctic rock, Svalbard
PP1	PRJNA418054	32	Terrestrial	Permafrost, Alaska
PP1	PRJNA430179	11	Ice/Snow	Glacier Ice/snow, Spain
PP1	PRJNA436954	8	Frehshwater	High-arctic microbial mat
PP1	PRJNA529498	33	Ice/Snow	Cryoconite hole, Antarctica
PP2	PRJEB11496	24	Marine	Marine sediment, Antarctica
PP2	PRJEB23054	59	Terrestrial	Permafrost, Alaska
PP2	PRJNA244335	31	Freshwater	Sediment and water from subglacial lake, Antarctica
PP2	PRJNA255432	70	Freshwater	Arctic lake, Canada
PP2	PRJNA278982	4	Marine	Ice-shelf water cavity, Antarctica
PP2	PRJNA321351	9	Freshwater	Arctic lake, Greenland
PP2	PRJNA324626	9	Terrestrial	Frozen soil, China
PP2	PRJNA430887	26	Ice/Snow	Glacier ice and weather crust, USA
PP2	PRJNA431087	1	Terrestrial	Microbial mat, Antarctica
PP2	PRJNA432743	7	Freshwater	Subglacial aquifer brine, Antarctica
PP2	PRJNA471245	51	Ice/Snow	Water, ice, soil, sediment and microbial mat, Antarctica
PP2	PRJNA480849	1	Ice/Snow	Cryoconite hole, Antarctica

PP2	PRJNA554442	11	Terrestrial	Permafrost, Alaska
PP2	PRJNA593264	13	Marine	Water, sediments and snow, Antarctica
PP2	PRJNA629965	6	Freshwater, Ice/Snow	Snow and glacier melt
PP2	PRJNA744712	72	Ice/Snow	Cryoconite hole, Antarctica

Supplementary Table 7. Summary of the cryospheric samples included in the two 16s rRNA amplicon datasets (primer pair 1 = PP1, and primer pair 2 = PP2).

Metric	Group	Comparison	Corr. p-value
Uniprot identity [%]	KEGG	Cryo.–Shared	2.9e-2
		Cryo.–Others	5.7e-10
		Shared-Others	1.2e-101
	Ambiguous	Cryo.–Shared	4.2e-3
		Cryo.–Others	2.6e-12
		Shared-Others	2.7e-87
	Unassigned	Cryo.–Shared	2.6e-7
		Cryo.–Others	2.1e-13
		Shared-Others	2.6e-3
Mean GC [%]	KEGG	Cryo.–Shared	2.7 e-5
		Cryo.–Others	1.1e-17
		Shared-Others	5.50e-292
	Ambiguous	Cryo.–Shared	7.8e-9
		Cryo.–Others	1.1e-50
		Shared-Others	4.20e-297
	Unassigned	Cryo.–Shared	8.6e-1
		Cryo.–Others	1.1e-68
		Shared-Others	0
Mean Cluster Identity [%]	KEGG	Cryo.–Shared	4.3e-19
		Cryo.–Others	2.1e-7
		Shared-Others	3.9e-81
	Ambiguous	Cryo.–Shared	3e-39
		Cryo.–Others	1.9e-11
		Shared-Others	3.9e-78
	Unassigned	Cryo.–Shared	8.8e-12
		Cryo.–Others	5.4e-1
		Shared-Others	5.4e-63

Supplementary Table 8. Unassigned functional clusters exact p-values for the pairwise two-sided Wilcoxon tests. Corrected p-values were adjusted using the Holm method. Sample sizes are listed in Table 1.
