# Supplementary material for: The microbiome of cryospheric ecosystems
Source: Nat Commun. 2022 Jun 2;13:3087. doi: 10.1038/s41467-022-30816-4 (PMC9163120; doi:10.1038/s41467-022-30816-4)
Supplement: Supplementary file 2 — Reporting Summary [file 41467_2022_30816_MOESM2_ESM.pdf]

nature portfolio

Corresponding author(s): Massimo Bourquin
Tom Battin

Last updated by author(s): May 10 2022

Reporting Summary

Nature Portfolio wishes to improve the reproducibility of the work that we publish. This form provides structure for consistency and transparency in reporting. For further information on Nature Portfolio policies, see our Editorial Policies and the Editorial Policy Checklist.

Statistics

For all statistical analyses, confirm that the following items are present in the figure legend, table legend, main text, or Methods section.

n/a	Confirmed	
☐	☒	The exact sample size (*n*) for each experimental group/condition, given as a discrete number and unit of measurement
☒	☐	A statement on whether measurements were taken from distinct samples or whether the same sample was measured repeatedly
☐	☒	The statistical test(s) used AND whether they are one- or two-sided<br>*Only common tests should be described solely by name; describe more complex techniques in the Methods section.*
☐	☒	A description of all covariates tested
☐	☒	A description of any assumptions or corrections, such as tests of normality and adjustment for multiple comparisons
☐	☒	A full description of the statistical parameters including central tendency (e.g. means) or other basic estimates (e.g. regression coefficient) AND variation (e.g. standard deviation) or associated estimates of uncertainty (e.g. confidence intervals)
☐	☒	For null hypothesis testing, the test statistic (e.g. *F*, *t*, *r*) with confidence intervals, effect sizes, degrees of freedom and *P* value noted<br>*Give P values as exact values whenever suitable.*
☒	☐	For Bayesian analysis, information on the choice of priors and Markov chain Monte Carlo settings
☒	☐	For hierarchical and complex designs, identification of the appropriate level for tests and full reporting of outcomes
☒	☐	Estimates of effect sizes (e.g. Cohen's *d*, Pearson's *r*), indicating how they were calculated

Our web collection on statistics for biologists contains articles on many of the points above.

Software and code

Policy information about availability of computer code

Data collection

The data was collected using the below listed methods.

16S rRNA Datasets.
Two primer pairs typically used in microbial ecology targeting the prokaryotic 16S rRNA were assessed: 341f-785r and 515f-806r. They will be referred to as Primer Pair 1 (PP1)13 and Primer Pair 2 (PP2)12, respectively. All articles citing the PP1 and PP2 reference articles were retrieved using Web of Science (All databases, searched on the 7th of December 2019, 1727 articles). Only studies having sequenced environmental samples were kept. Simultaneously, studies assessing pollution or contamination and involving major climatic or ecological events, e.g. storms or blooms, were removed. Thereafter, a second selection was performed based on the whole article, assessing technical criterions. Only studies using the aforementioned primer pairs, Illumina paired-end sequencing and having available data were kept; and the corresponding NCBI bioproject accessions were extracted. At a later stage, a few studies meeting the filtering criteria but not included in the Web of Science search were added. The raw sequencing (fastq) data were downloaded using the ENA browser (European Nucleotide Archive; www.ebi.ac.uk/ena/browser/). At this stage, only the control samples were downloaded for experimental studies.

Data analysis

16S rRNA analyses.
The read files were filtered as follows: First, Trimmomatic was used to remove low quality reads, truncating the reads at the first instance of a sliding-window (5bp) having a mean quality below 15. At this stage, the raw data from each BioProject was imported into qiime2. Denoising was performed with the dada2 plugin using the primers sequences length for the '–p-trim-left-r' and '–p-trim-left-f' parameters45. This step removed integrally two studies in the PP1 dataset ("negative values in quality" and "all samples discarded" errors). Only sequences assigned to bacterial taxa were kept, and chloroplast and mitochondrial sequences were also removed. Finally, all samples with less than 5000 reads after this initial filtering were removed.

Taxonomy classification for PP1 and PP2 ASVs was performed using the qiime2 'feature-classifier' plugin and the Silva 138 nr database. First, reads were extracted from the reference sequences using the extract-reads method. For this, the primer sequences were used for the '–p-r-

primer' and '–p-f-primer' arguments. The length of the extracted reads was set to min. 250 and max. 450 for the PP1 dataset and min. 200 and max. 400 for the PP2 dataset. A classifier was then created using the fit-classifier-naïve-bayes method with the extracted reads and the reference taxonomy. Finally, this classifier was run on the dataset's sequences using the 'classify-sklearn' method to get the sequences taxonomy4. To keep only high-quality samples, all samples having less than 75% of their ASVs assigned to the phylum level, and 50% assigned to the genus level were removed. This filtering resulted in 2508 samples and 530,254 ASVs for PP1 and 1739 samples and 410,931ASVs for PP2. The ASV tables and metadata tables for these datasets can be found on Zenodo, under the file names: 'Data/PP1_table.tsv', 'Data/PP2_table.tsv' and 'Metadata/PP1_metadata.tsv' and 'Metadata/PP2_metadata.tsv', respectively.

Metagenomic dataset. To address the functional aspect of identified taxa, accession numbers of studies comprising of the following keywords: metagenomics, whole genome shotgun, and environmental, were queried using NCBi's EDirect (v1.1). The results were manually curated to select studies from a broad Geographic distribution, yielding a total of 382 datasets. The selection of metagenomic samples was further restricted to raw fastq data, thus precluding the use of samples from MG-RAST since only the metagenome assembly files were provided. Additionally, all samples still under embargo in accordance with the Joint Genome Institute (JGI; USA) policy, were excluded. From this collection, samples with fewer than 1 million reads or with a quality of reads less than Q25 were removed for a final collection of 91 samples (Fig. 1A). Paired reads were processed using the Integrated Meta-omic Pipeline (IMP; v2.0). The workflow includes pre-processing such as primer/adapter removal and trimming followed by an iterative assembly. Additionally, functional annotation of genes based on custom databases was performed (described below). The entire workflow is setup in a reproducible Snakemake (v5.16) format. Briefly, after preprocessing the reads, de novo assembly using MEGAHIT (v1.2) assembler was performed. The metagenomic dataset KEGG Orthologs (KO) table, taxonomy table, and metadata are available on Zenodo under the 'Data/MTG_KEGG_counts.tsv', 'Data/MTG_table.tsv', and 'Metadata/MTG_metadata.tsv'.

Metagenomic taxonomic classification and functional analyses. Functional potential analyses from contigs were determined by predicting open-reading frames using a modified version of Prokka (v1.14.5) including Prodigal (v2.6.3) gene predictions for complete and incomplete open reading frames. Genes identified subsequently were annotated with Hidden Markov Models (HMM; v3.153), trained using an in-house database. The annotations were further annotated with KO55 groups using 'hmmsearch' from HMMER 3.153. Upon multiple functional group assignments, the best hits based on bit scores were selected. FeatureCounts56 with the '-p' and '-O' arguments were then used to extract the number of reads per functional category.

Logistic regression classification of cryospheric bacterial communities. The Logistic regression implemented in scikit-learn python module (v0.23.2) was trained on presence-absence ASV tables to classify cryospheric samples. To reduce the amount of ASVs considered, the table was filtered based on relative abundance: presence was defined at a 0.005 relative abundance threshold. A 10-fold cross-validation (CV) was ran and balanced accuracy was averaged across the CVs. The C parameter controlling the L2 penalisation was optimized testing 30 values linearly distributed between 0.01 and 0.5, the one with the best balanced accuracy (averaged values of 5 random iterations of 5-fold cross validation, means of PP1 and PP2 values were averaged) was selected (C= 0. 178966). ROC curves were plotted using the 'plot_roc_curve' function of the scikit-learn python module. Balanced accuracy, precision and recall were computed using the 'accuracy_score', 'precision_score' and 'recall_score' methods, respectively, with sample weights correcting for the sample size of the cryospheric and non-cryospheric datasets(Supplementary Table 1). The different accuracy metrics values for the classifiers can be found in table S1. Odds ratios were calculated using the exponent of the logistics models' coefficients. The tables containing the ASVs logistic regressions odds ratios can be found in the Data folder available on Zenodo under the name 'PP1_Logistic_coefs.csv' and 'PP2_Logistic_coefs.csv' for PP1 and PP2, respectively.

Phylogenetic analyses. Phylogenetic trees were built using the set of ASVs found in the dataset used for the logistic regression classification. Due to the different 16S regions targeted, phylogenies for both PP1 and PP2 datasets were constructed separately. The ASVs sequences were aligned using the FFT-NS-2 algorithm implemented in the Mafft (v7.0) aligner with default parameters. The alignments were subsequently trimmed using TrimAl (v1.3) with the '-gt 0.95' parameter, and the trees built using IQ-TREE (v1.6.12) with the GTR model of nucleotide substitution and the '-fast' option. Phylogenetic tree visualisations were created using the ggtree (v3.15) and ggtreeExtra R (v3.6) packages. Only positive coefficients showing enriched presence in cryospheric environments are shown in the phylogenetic barplots (Fig. 1). The number of ASVs with an odds ratio above 1 was shown for taxonomic summaries.

ß-diversity phylogenetic metrics (Sorensen's Index and ß-MNTD) were computed using the 'phylosor' and 'comdistnt' functions of the Picante (v1.8.2) R package, using custom functions to compute pairwise comparisons. For each metric, 50 iterations were performed where we calculated the pairwise distances between and within 50 cryospheric, and 50 non-cryospheric samples. This random sub-sampling approach was chosen to reduce computing time. Kruskal-Wallis tests were used to determine whether the distribution was different across groups, and Wilcoxon tests were used to calculate pairwise post-hoc comparisons. Wilcoxon tests implemented in the compare_means function of the ggpubr R package were used, effects sizes (r) were calculated with the wilcox_effsize function implemented in the statix R package. Sample specific calculations of ?-PD (and species richness),-MPD and-MNTD were computed using the 'pd', 'mpd' and 'mntd' functions of the Picante R package63. Linear models were used to compare the values of-PD,-MPD and-MNTD across samples, taking the logarithm of the species richness and the dataset (PP1 and PP2) as a fixed effect.

Differential abundance analysis. Using the Silva Taxonomic information, ASV raw counts were aggregated to the genus-level using a custom R script, removing the ASVs not assigned taxonomically to the genus-level. Ancom (v2.1) was used on the count data for the differential abundance analysis, using the default W statistic threshold of 0.764. The 'zero-cut' parameter was set to 0.995 to consider all bacterial genera present in at least 21 samples (n = 4247), and the primer pair (PP1 and PP2) variable was taken as a random effect with the rand_formula parameter ("~ 1 | Dataset"). We considered as significantly enriched genera (i.e. cryospheric genera), the ones with a W statistic above the threshold (0.7) and a positive value of CLR mean difference. GGplot2 (v2.15) was used to modify the Ancom figure showing the results of the differential abundance analysis. The 'heat_tree' function of the metacoder R package (v0.3.4) was used to show the number of cryospheric bacterial genera, at various taxonomic level, using taxonomic trees. The results of this analysis can be found in the Data/ folder available on Zenodo under the name 'Ancom_amplicon_res.csv' file.

NCBI Refseq genomes properties. To assess the genome size and GC content of publicly available prokaryote genomes, a non-redundant list encompassing all the genera in our datasets was compiled. Thereafter, the list of prokaryote genomes (prokaryotes.txt) available on NCBI was downloaded on March 15th, 2021 from https://ftp.ncbi.nlm.nih.gov/genomes/GENOME_REPORTS. The prokaryote list was filtered based on the list of genera found in our dataset, simultaneously retrieving the accession IDs. The accession IDs were used to download the complete bacterial genome sequences using the ncbi-genome-download (v1.0) python package (https://github.com/kblin/ncbi-genome-download). The

genome sizes for the downloaded genomes were additionally retrieved from the prokaryotes.txt metadata file. Subsequently, Prodigal was used to annotate the open-reading frames per genome obtaining both the general feature format (gff) files and aminoacid fasta (faa). These were used thereafter as input used to estimate the predicted growth time (in hours) and their codon usage analyses (CUB) using gRodon (v1.0) and coRdon (v1.15) (https://github.com/BioinfoHR/coRdon) R package respectively. The amino acid enrichment analysis was performed on by converting the codon counts to amino acids using the R-package Biostrings using DEseq2 (v2.3) with default parameters (log-median ratio normalization across genera). Wilcoxon tests implemented in the compare_means function of the ggpubr R package were used, effects sizes (r) were calculated with the wilcox_effsize function implemented in the statix R package. The relevant scripts and information for these analyses are openly available and included in the code availability section. The corresponding files used for this analysis can be found in the Data/ folder available on Zenodo under the names 'prokaryotes.txt', 'merged_all_codon_counts.txt' and 'merged_all_growth_prediction.txt'.
Structure of the cryospheric microbiome. Non-metric multidimensional scaling was used to visualise the composition of cryospheric bacterial communities according to the ecosystem types and primer pairs. For this, the 'metaMDS' function implemented in the package vegan was used with Bray-Curtis distances. The stress for the chosen value of k=2 was 0.206. The 'adonis2' function was used to perform a PERMANOVA analysis to test the effect of the ecosystem type and the primer pairs on the composition of bacterial communities (Supplementary Table 4). Pairwise comparisons between ecosystem types were tested using the function 'pairwise.adonis2'. P-values were adjusted using the default Bonferroni method, to account for multiple comparisons.

The prevalence of each genus was modeled as the probability of presence using a logistic binomial regression (with the R stats 'glm' method), using the ecosystem type (snow/Ice, terrestrial, marine and freshwater) and the primer pair as fixed effect. To calculate the probability of occurrence in the cryosphere for each genus, the prediction was calculated for all ecosystem types and primer pair combinations, and averaged. The core microbiome was defined at 0.1% abundance, and 20% prevalence across the cryosphere, for genera present in at least one sample in all four ecosystem types (Supplementary Figure 2B). The core microbiome presence in the different ecosystem types was shown using an upset-plot using the complex-upset (v.1.3.3) R package. The taxonomic tree available in Supplementary Figure 2A was created using the Metacoder R package. The α-diversity was calculated using Shannon's index with a custom R functions. To test the difference across ecosystems and datasets, the Wald-Type statistics implemented in the 'GFD' function of the R GFD package was used (Supplementary Table 5). This test was performed instead of an ANOVA, as the data was not normally distributed. The mean values given by the function were used for the ecosystem comparison.

KEGG enrichment. The standard DESeq2 pipeline with default parameters was used on raw KEGG counts for the enrichment analysis, using the default Wald tests. We considered significantly enriched Kegg Orthologs (KOs) with an FDR adjusted p < 0.01 and a log2 fold-change > 1. To unravel the contribution of these gene families to functional pathways, we ran KEGGdecoder (v1.3) on the KOs enriched in cryospheric samples, to identify environmental-associated pathways in all samples.

To understand and unravel the origins of the gene families specific to the cryospheric metagenomes, contigs were taxonomically classified following which the specific gene families were mapped to the contigs. We used Kraken2 (v2.14) to taxonomically assign all the contigs present in the metagenomes followed by custom python scripts (provided) to link the genes belonging to enriched KEGG orthologs (KO and the corresponding NCBI taxon ID. An R script using the NCBI entrez package was used to retrieve the taxonomy based on the taxon ID, and to get the genus-level taxonomy. To link the Silva genus taxonomies with their NCBI counterparts, the grep function included in R allowing partial matches was used to find Silva genera name matching the NCBI genus name. The DEseq2 results, KEGG-decoder output and taxonomy matches can be found in the Data/ folder of the Zenodo under the names 'KEGG_deseq_results.csv', 'KEGG_decoder_output', and 'KEGG_sign_tax_genera.csv', respectively.

Gene clusters and unassigned protein coding sequences. Predicted gene sequences annotated to the KEGG database and those unassigned were gathered into individual groups based on KEGG ID or Unassigned using a custom python script. 'annotation2gene.py'. The fasta files were subsequently concatenated and clustered to identify functional homologs within the dataset. We used mmseq2 (v13-4511) 'linclust' to cluster the 41,068,842 gene sequences found in the entire metagenomic dataset. Subsequently, fasta sequences associated with each cluster were retrieved into separated clusters (n=12,125) and linked to the coverages to estimate abundances. MAFFT was then used to create a multiple sequence alignment of the sequences per cluster, while the 'cons' method from EMBOSS (v.2.0.0) was used to generate a consensus sequence. The generated consensus sequences from each cluster were subsequently annotated and their identity verified against the UniProt TrEMBL (release 26.0) database. The pairwise identity of sequences within each cluster was measured using CLUSTAL (v2.0) 'distmat' option with the '–percent-id'. Wilcoxon tests implemented in the compare_means function of the ggpubr (v0.4.0) R package were used, effects sizes (r) were calculated with the wilcox_effsize function implemented in the statix (v1.3.0) R package. The unassigned clusters summary statistics and Uniprot matches can be retrieved on Zenodo, in the Data/ folder under the names 'Unassigned_clusters_stats.tsv', and 'unassigned_uniprot_matches.txt'.

Code availability: All scripts used for analyses, along with the conda environments, and additional information is provided in a Github repository archived on Zenodo: https://zenodo.org/badge/latestdoi/371641303.

For manuscripts utilizing custom algorithms or software that are central to the research but not yet described in published literature, software must be made available to editors and reviewers. We strongly encourage code deposition in a community repository (e.g. GitHub). See the Nature Portfolio guidelines for submitting code & software for further information.

Data

Policy information about availability of data

All manuscripts must include a data availability statement. This statement should provide the following information, where applicable:

- Accession codes, unique identifiers, or web links for publicly available datasets
- A description of any restrictions on data availability
- For clinical datasets or third party data, please ensure that the statement adheres to our policy

Data availability
The data is fully available on Zenodo at https://doi.org/10.5281/zenodo.6325482

Databases used in the current study include the following:

1. maxikraken database: https://lomanlab.github.io/mockcommunity/mc_databases.html
2. SILVA database: https://www.arb-silva.de/documentation/release-1381/

Field-specific reporting

Please select the one below that is the best fit for your research. If you are not sure, read the appropriate sections before making your selection.

☐ Life sciences ☐ Behavioural & social sciences ☒ Ecological, evolutionary & environmental sciences

For a reference copy of the document with all sections, see nature.com/documents/nr-reporting-summary-flat.pdf

Ecological, evolutionary & environmental sciences study design

All studies must disclose on these points even when the disclosure is negative.

Study description	The study consists of a meta-analyses of several published 16S rRNA and metagenomic sequencing studies pertaining to the cryosphere. Each sample was treated an individual data point, with at least 5 samples per group for the bulk genomic properties analyses.
Research sample	Publicly available sequencing data spanning various sample types, including but not limited to snow, ice, polar oceans, freshwater sediments, deserts, volcanic sediments etc. were collected from the European Nucleotide Archive (ENA). The samples were chosen so as to represent a broad range of habitat and ecosystems types.
Sampling strategy	Since publicly available data was used for this study, the sampling strategy is not applicable.
Data collection	Since publicly available data was used for this study, the data collection was restricted to downloading publicly available data from ENA. This has been described above in the "Data Collection" section.
Timing and spatial scale	Since publicly available data was used for this study, this section is not applicable.
Data exclusions	Samples with fewer than 1 million reads were excluded from the analyses for the shotgun metagenomes, since meaningful estimates of taxonomy and/or abundances would not be possible. For 16s rRNA amplicon data, only sequences assigned to bacterial taxa were kept, and all samples with less than 5000 reads after this initial filtering were removed. To keep only high-quality samples, all samples having less than 75% of their ASVs assigned to the phylum level, and 50% assigned to the genus level were removed.
Reproducibility	To ensure maximum reproducibility, the necessary methodology including the code for the extensive data analyses and figure generation are provided. All the code is fully available via Github.
Randomization	Since publicly available data was used for this study and it was a meta-analyses based on specific habitats and ecosystems, this section is not applicable.
Blinding	Since publicly available data was used for this study, this section is not applicable.

Did the study involve field work? ☐ Yes ☒ No

Reporting for specific materials, systems and methods

We require information from authors about some types of materials, experimental systems and methods used in many studies. Here, indicate whether each material, system or method listed is relevant to your study. If you are not sure if a list item applies to your research, read the appropriate section before selecting a response.

Materials & experimental systems

n/a	Involved in the study	
☒	☐	Antibodies
☒	☐	Eukaryotic cell lines
☒	☐	Palaeontology and archaeology
☒	☐	Animals and other organisms
☒	☐	Human research participants
☒	☐	Clinical data
☒	☐	Dual use research of concern

Methods

n/a	Involved in the study	
☒	☐	ChIP-seq
☒	☐	Flow cytometry
☒	☐	MRI-based neuroimaging
